# Soil and plant communities co-regulating plant biomass allocation patterns along a saline-alkali gradient, case study of *Allium ramosum* in Songnen Grassland, Northeast China

**DOI:** 10.3389/fpls.2025.1627304

**Published:** 2025-09-24

**Authors:** Changxing Fu, Jiayan Fan, Gaohua Fan, Heqi Wang, Congwen Wang, Wei Qiang, Dafu Yu, Yingxin Huang

**Affiliations:** ^1^ State Key Laboratory of Black Soils Conservation and Utilization, Northeast Institute of Geography and Agroecology, Chinese Academy of Sciences, Changchun, China; ^2^ University of Chinese Academy of Sciences, Beijing, China

**Keywords:** life history strategies, biomass allocation, allometric, soil salinity, perennial herbs

## Abstract

**Aims:**

Plants have developed sophisticated mechanisms to adapt to changing environments. The strategies for biomass allocation is critical for plant to ensure fitness. Increasing soil salinity has a dramatic impact on plant growth and reproduction from the individual to community level. However, our understanding of how plants adapt their biomass allocation strategies to changes in soil salinity and community structure is incomplete.

**Methods:**

We investigated 122 individuals of perennial herb *Allium ramosum* from 70 plots along a soil gradient in Songnen grassland. Field investigations determined the physicochemical properties of the soil, aboveground biomass, richness and individual and each organ (root, stem, leaf, flower, and bulb) biomass of A. ramosum.

**Results:**

The results showed that plant community aboveground biomass and community-weighted height decreased as soil salinity increased. *A. ramosum* individual size decreased, the allometric exponents between reproductive organs (flowers) and storage organs (bulbs) also decreased, with more biomass allocated to flowers. However, this trend was indirectly influenced by salinization through a reduction in community weighted height (reducing light competition) and community aboveground biomass (altering competitive pressure for resources), rather than a direct response to soil salinity.

**Conclusions:**

This study highlights the complex interplay between community structure and individual plant adaptation strategies in response to environmental gradients, emphasizing the role of community-scale processes in regulating individual resource allocation.

## Introduction

Plants maintain population survival and development by adjusting life history strategies, including nutrient acquisition and reproductive strategies (Bonser, 2013; Zemunik et al., 2015; Wong et al., 2024). Changes in such strategies vary depending on the environment and life history of different plant populations (Planas-Sitja et al., 2023; Van de Walle et al., 2023; Stott et al., 2024). Biomass allocation patterns reflect the trade-offs plants make between growth, reproduction, and survival, as total resources for these functions are limited (Poorter et al., 2012; White et al., 2022). When resources are limited, plants may prioritize their growth and reproduction strategies to ensure population survival. For example, in low-light conditions, plants may grow taller with increased stem biomass to capture more light, while in nutrient-poor soils, they may allocate more biomass to roots to enhance nutrient uptake (Xiao et al., 2021; Bartuskova et al., 2022). Thus, changes in soil characteristics may have a dramatic impact on plant life history strategies. Soil salinity degradation is a common type of soil degradation, which is exacerbated by climate change and anthropogenic impacts (Cao et al., 2021; Corwin, 2021; Garcia-Franco et al., 2021). Soil salinization results in elevated soil pH, while soil nutrient content decreases. Further restrict plant transpiration by decreasing soil water potential or have a toxic effect on plant roots due to high osmotic pressure (Li et al., 2022; Liu et al., 2024).

Consequently, empirical evidence indicates that fluctuations in soil salinity induce structure and functional modifications within plant communities (Cao et al., 2021; Heng et al., 2022; Xiao et al., 2025). Given that species belonging to diverse families and functional groups exhibit a spectrum of phenotypic plasticity in response to environmental gradients, this response often leads to shifts in population dynamics and subsequent alterations in ecosystem processes (Zhou et al., 2021; Van de Walle et al., 2023; Beccari and Carmona, 2024; Wu et al., 2024). It is especially critical to understand how plants adapt to soil salinization and community changes by adjusting biomass allocation to construct new leaves, stems, and roots in order to maintain growth and reproduction (Chen et al., 2021; Bartuskova et al., 2022; Dolezal et al., 2024). However, our understanding of how plants adapt to biotic and abiotic factors by adjusting their life history strategies to maintain populations in different communities remains limited. The trade-off between growth, nutrient acquisition and reproduction derives from the fact that when limited resources are allocated to one function (e.g., reproduction), they are not available for others (Weiner et al., 2009; Skarpaas et al., 2016; Umana et al., 2021). Some species may allocate more resources to growth and nutrient acquisition to maintain their survival, while others may prefer to allocate resources to increase the number of offspring, reflecting a coordinated allocation of biomass among organs due to variation in life history strategies (Bonser, 2013; Fujita et al., 2014; Bartuskova et al., 2022). For example, in nutrient-rich environments, plants may have easier access to nutrients and thus more energy for reproduction, whereas in situations where resources are scarce, plants may prioritize securing growth but not reproduction (Hulshof et al., 2012; Hu et al., 2021; Fang et al., 2023; Dolezal et al., 2024). The duration of plant life history and reproductive patterns can also influence the trade-off between nutrient acquisition and reproduction (Weiner et al., 2009; Stott et al., 2024). Typically, annual species usually concentrate their growth and reproduction in a short period, whereas perennial plants can acquire nutrients and produce seeds over multiple growing seasons (Hu et al., 2021; Fang et al., 2023).

However, the West, Brown, Enquist (WBE) model suggests that the approximate fractal structure of vascular plants allows the parts of individuals to grow in a specific proportion, which is not isometric but rather allometric (West et al., 1999; Zhang et al., 2021b). For instance, there is an allometric exponent of 3/4 observed between stem-leaf and root-leaf biomass (Enquist and Niklas, 2002). Similarly, an allometric relationship exists between reproduction and aboveground biomass (Weiner et al., 2009). Studies on biomass allocation patterns in various plants revealed that the same allometric relationships were present (Tang et al., 2022). In other words, plants differing in communities and functions exhibited uniform allometric relationships (Enquist and Niklas, 2002). Recent studies have found that changes in environmental conditions can alter the allometric exponent among plant organs, indicating that the pattern of plant biomass partitioning is modified (Zhang et al., 2021b; Tang et al., 2022; Zhang et al., 2022). Soil nutrient gradients were significantly correlated with stem and leaf allocation of biomass at the community level, with nutrient-enriched environments promoting high allocation of stems to compete for light, whereas in infertile habitats communities tended to allocate more biomass to leaves than stems (Yan et al., 2016). Similar results also reported in nutrient-rich forests, where species tend to increase stems (Kim et al., 2020). In addition, plants adapted to phosphorus-limited habitats were found to tend to reduce resource allocation for sexual reproduction (Fujita et al., 2014). The existing studies have primarily concentrated on the direct effects of soil nutrient content on plant life history strategies, while neglecting to consider the impact of soil-dependent plant communities on these life history strategies. Furthermore, numerous studies have examined interspecific variation in plant strategies, yet these have scarcely addressed how the life history strategies of a particular species vary with soil and community characteristics (Fujita et al., 2014; Yan et al., 2016; Dolezal et al., 2021; Fang et al., 2023; Tsogtsaikhan et al., 2025).

The *Allium ramosum* L. is a perennial herb belong to the *Liliaceae*. It is found in grasslands of northern China, where it frequently occurs as a component of communities with other species (Ge et al., 2020). *Allium* L. species are characterized by the presence of a bulb, which functions as an underground storage organ (Chope et al., 2012; Hsiao et al., 2019). The physiological and ecological functions of bulbs are of significance to the life cycle of plants (Ashagrie et al., 2021). They are the primary nutrient storage organ, storing substantial amounts of starch, sugar, and other nutrients (Howard and Cellinese, 2020). These stored nutrients provide energy and nutrients during plant growth and under unfavorable conditions, thereby facilitating survival and growth (Hsiao et al., 2019). Thus, utilizing *Allium ramosum* as a model species enables us to more directly investigate the trade-offs between growth, storage, and reproduction in plants.

In the present study we focused on plant biomass allocation patterns and investigated whether they were affected not only by soil features but also by plant community structure along an soil gradient in the natural grassland. We hypothesized that 1) along the soil gradient the biomass allocation strategy of *Allium ramosum* is plastic, e.g. wild leeks allocate more biomass to reproductive organs (flowers) at sites with heavier soil salinization and more biomass to growth and nutrient storage organs (leaves, bulbs) at sites with lighter soil salinization; 2) The altered biomass allocation strategy of *Allium ramosum* was not influenced by soil characteristics alone (N and P content), but by a combination of soil and plant community characteristics.

## Materials and methods

### Study site and plant sampling

The study was conducted at the Changling Grassland Research Station of the Chinese Academy of Sciences, located in Jilin Province, on the southwestern part of the Songnen Plain. Geographically, the site is positioned at 121°30′-123°44′-E, 44°44′-48°40′N, situated in the eastern part of the Inner Mongolian Plateau. The altitude of the area ranges from 138 to 145 meters above sea level. Since 2001, the site has been fenced to prevent grazing, thereby minimizing its impact on the vegetation (Huang et al., 2016). The Songnen grassland, situated near the Mongolia Plateau, experiences a typical continental semi-arid monsoon climate. The study area receives an average annual rainfall of approximately 470 mm, which varies significantly from year to year. In contrast, the annual evaporation rate exceeds 1,500 mm, more than three times the average annual precipitation. The mean annual temperature hovers around 4.9 °C, and the frost-free period lasts between 120 and 150 days. Characterized as a typical meadow grassland, the plant community is predominantly composed of *Stipa baicalensis* in areas with lower soil salinization. However, the vegetation is complex and diverse due to the influence of soil salinity. In more saline areas, the dominant species are mainly salt-tolerant graminoids such as *Leymus chinensis* and *Puccinellia tenuiflora*, and *Suaeda glauca*, which appear in the area of heavier salinity (Huang et al., 2019).

### Plant traits and soil feature measurements

We selected 7 community types along the soil gradient (**Table 1**), and ten 1 m×1 m plots were selected for each community. Within each plot, 1–2 *A. ramosum* plants were randomly selected. Thus,10–20 individuals were taken from each community. A total of 122 individuals of *Allium ramosum* from 70 plots were sampled.

**Table 1:** Community and soil features of each site.

Site	Dominant species	Community			Soil				
		A-biomass	CWM-height	Richness	SOC	pH	EC	TN	TP
1	*Pocockia ruthenia*	518.16±68.47a	33.57±5.11a	13.0±2.21a	14.72±2.42a	8.539±0.14a	139.27±11.01a	1799.53±265.75a	376.01±40.06a
2	*Calamagrostis epigeios*	417.56±45.87b	27.36±4.17b	14.1±1.72a	12.01±2.55b	8.647±0.08a	148.06±18.34a	1500.03±213.06b	340.53±63.82ab
3	*Lespedeza bicolor*	346.71±40.33c	26.76±3.93b	13.5±2.17a	10.88±1.22b	8.667±0.04a	154.17±19.00a	1424.60±231.48b	304.40±46.09bc
4	*Leymus chinensis*	302.56±51.34c	27.03±4.31b	11.0±2.44ab	12.04±2.08b	8.696±0.08a	162.18±23.07a	1276.69±232.48bc	301.87±49.31bc
5	*Aster pekinensis*	234.15±26.83d	26.67±3.73b	12.0±2.94ac	9.89±1.19b	8.763±0.11a	178.37±17.12a	1099.05±145.93cd	288.61±39.11bc
6	*Aeluropus sinensis*	199.18±42.74de	26.48±2.82b	9.2±2.69bc	6.71±0.60c	9.252±0.56b	262.56±113.17b	940.72±169.98de	271.27±37.24c
7	*Chloris virgata*	153.20±37.68e	19.98±3.58c	8.8±1.98b	2.71±0.87d	9.886±0.17c	421.45±92.51c	760.13±108.95e	261.57±21.89c

SOC, soil organic carbon, TN, total nitrogen, TP, total phosphorus. Significant differences are indicated by lower case letters.

For each *Allium ramosum* individuals from different communities, we measured the height and separated the organs into roots, stems, leaves, flowers and bulbs. They were weighed after drying at 65 °C for 48 hours. At the same time, we took three soil samples at 0–15 cm depth in each plot and pooled them as a composite sample to measure soil nutrients. Some of the sieved samples were used to quantify available nutrients. Soil pH and electrical conductivity (EC) were measured using pH meter and conductivity meter in a soil:water suspension (1:5) after 5 min of shaking, respectively. We used potassium dichromate oxidation external heating method to determined soil organic carbon (SOC) content. The total nitrogen content (TN) and total phosphorus (TP) was measured by spectrophotometry. Soil available P was extracted by NaHCO_3_ and determined by Molybdate colorimetric method (Murphy and Riley, 1962). Available N was determined by the alkali diffusion method.

### Data analysis

The assessment of soil fertility based on individual soil nutrients may not be comprehensive, as the co-limitation of multiple nutrients is a common occurrence. Consequently, we employed principal component analysis (PCA) to condense the complexity of soil nutrients (soil available nitrogen, phosphorus, organic carbon, total nitrogen, total phosphorus, and the N/P ratio) into fewer dimensions using the ‘psych’ package. The use of multiple unrelated nutrients in PCA may reduce the explanatory power of the first axis, but the principal components along the axes better capture nutrient variation along a soil gradient. To select an appropriate axis for the soil nutrient gradient, we also analyzed the correlations between the first four principal axis and each soil indicator.

Community-weighted means were used to describe the height of the plant community.

For each species in the community, we randomly selected five plants to measure height, and subsequently calculated plant community-weighted heights based on the ratio of species biomass in the community.

Coefficients of variation were used to describe the stability of plant communities and species as soil salinity increased. Standard principal axis regression analyses were used to obtain coefficients of biomass partitioning among *Allium ramosum* organs by ‘smatr’ package, and linear regression models were used to analyze the relationships between soil principal components and community and *Allium ramosum* traits. Finally structural equation modelling was used for analyzing the direct and indirect effects of soil salinity on plant biomass allocation by ‘lavaan’ package. All analyses were carried out in R 4.2.0.

## Results

### Soil gradients in the natural grassland

**Figure 1:**
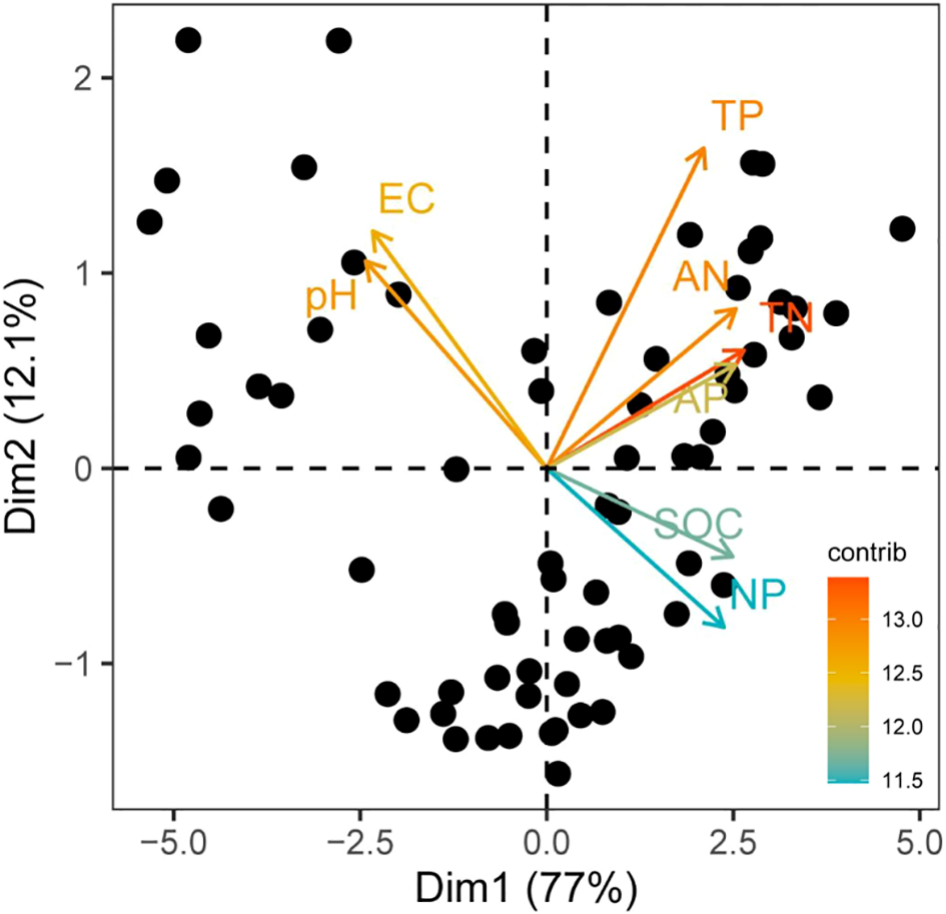
Principal component analysis (PCA) of soil nutrients. SOC, soil organic carbon; TN, total nitrogen; TP, total phosphorus; AN, available nitrogen; AP, available phosphorus; NP, nitrogen-phosphorus ratio.

**Figure 2:**
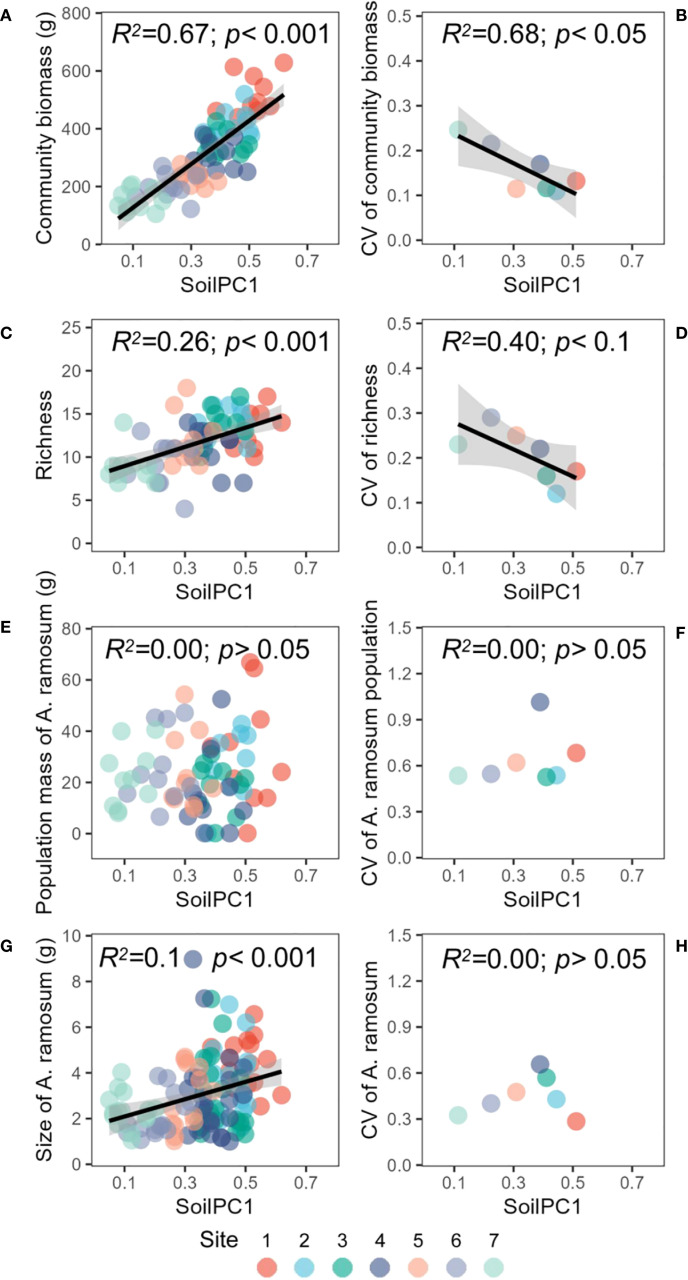
Relationships between the soil gradient (PC1) and **(A)** community above ground biomass, **(B)** CV of community above ground biomass, **(C)** richness, **(D)** CV of richness, **(E)** population mass of *A. ramosum*, **(F)** CV of *A. ramosum* population mass, **(G)** size of *A. ramosum*, **(H)** CV of *A. ramosum* size. The colors of the points represent different sites (dominant species of the plant community).

Soil salinity, as indicated by pH and electrical conductivity (EC), demonstrated an inverse relationship with nutrient content. Specifically, an increase in soil salinity corresponded to a decrease in soil organic carbon (SOC) content and overall nutrient availability (**Figure 1**). The first principal axis explained 77% of the variation in soil salinity and nutrient indicators (**Figure 1**). The pH and EC exhibited a negative correlation with the first principal axis, while SOC, total nitrogen (TN) and other nutrient indicators exhibited a positive correlation with the first principal axis (**Supplementary Table S1**). Consequently, the first principal axis can be interpreted as a representation of the soil salinity gradient, ranging from high salinity and low nutrients to low salinity and high nutrients. A significant and positive correlation was identified between plant community above-ground biomass and soilPC1, suggesting that as soil salinity decreased and nutrients increased, plant community biomass and stability increased (**Figures 2A, B**). The trend of species richness and community above-ground biomass exhibited consistency (**Figures 2C, D**). Soil salinity did not alter the relationship between plant community biomass and diversity; however, species evenness demonstrated a significant decrease as plant community aboveground biomass increased (**Supplementary Figure S1**). The *Allium ramosum* population biomass and stability remained relatively unresponsive to variations in soil gradient, although the size of individual *Allium ramosum* plants exhibited an increasing trend with increasing soil gradient (**Figure 2E, F**). However, the variability in individual size did not demonstrate a significant linear relationship with soil gradient (**Figure 2H**). Concurrently, the height of individual *Allium ramosum* plants and the biomass of each organ increased with soil gradient, particularly the aboveground biomass. Notably, the flower biomass exhibited no significant change with soil gradient (**Supplementary Figure S2**).

### Changes in biomass allocation patterns across soil gradients

**Figure 3:**
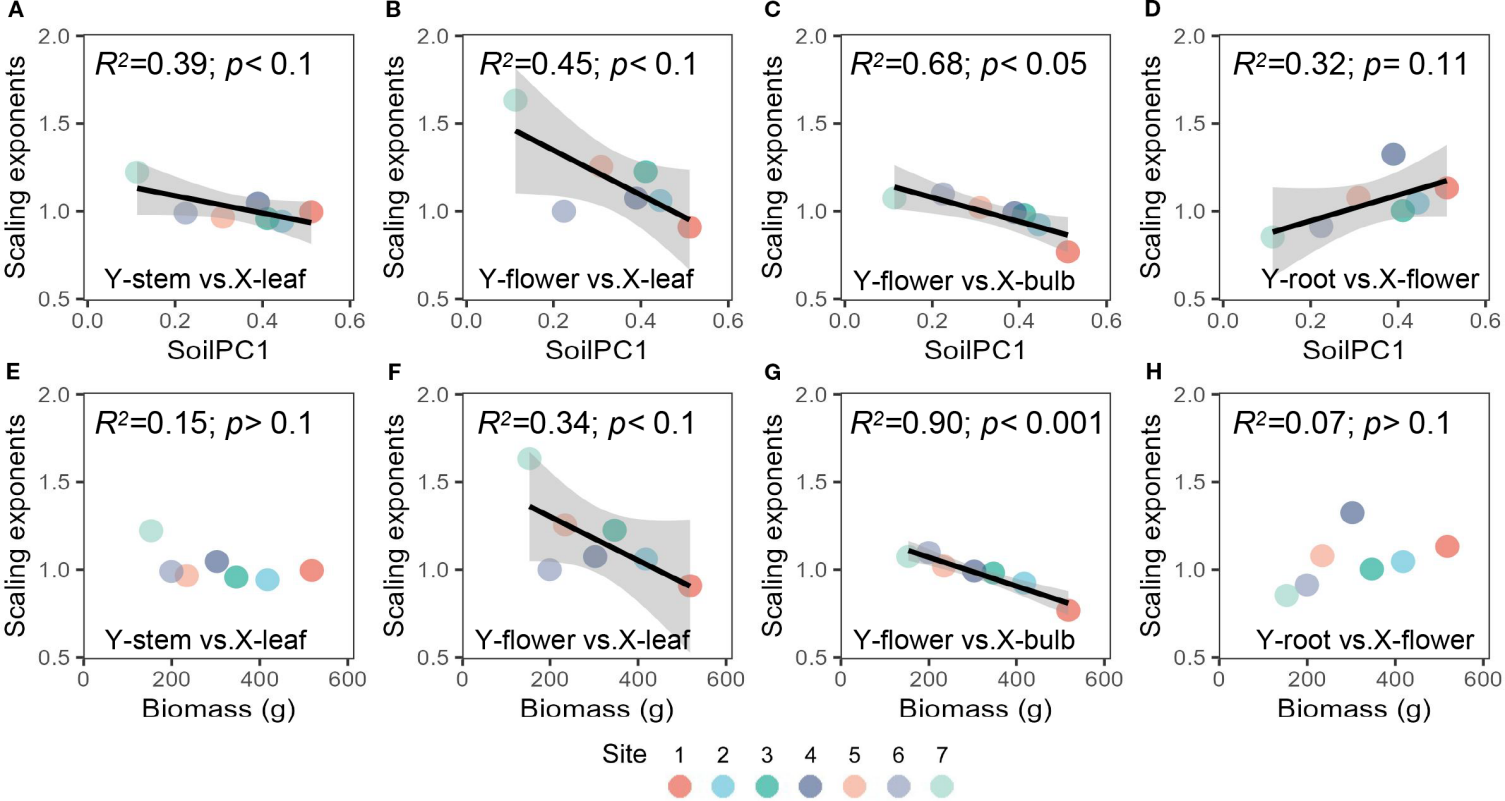
Relationships between the soil gradient (PC1) and scaling exponents of **(A)** leaf *vs* stem, **(B)** leaf *vs* flower, **(C)** bulb *vs* flower **(D)** flower *vs* root, and relationships between community above-ground biomass and **(E)** leaf *vs* stem, **(F)** leaf *vs* flower, **(G)** bulb *vs* flower **(H)** flower *vs* root. The colors of the points represent different sites (dominant species of the plant community).

With soil gradient, the allometric exponent between leaf and flower biomass and between bulb and flower biomass decreased (i.e. plants tended to allocate more biomass to flowers relative to plant growth and storage organs (leaves, bulbs)) (**Figures 3C, G**). Similarly, the allometric exponent between leaves and stems decreased with soilPC1 (more biomass was allocated to stems) (**Figure 3A**). Significant allometric partitioning among other organs of *Allium ramosum* existed, but did not vary significantly with soil gradients or plant community aboveground biomass (**Supplementary Table S3**, **Supplementary Figure S3**).

**Figure 4:**
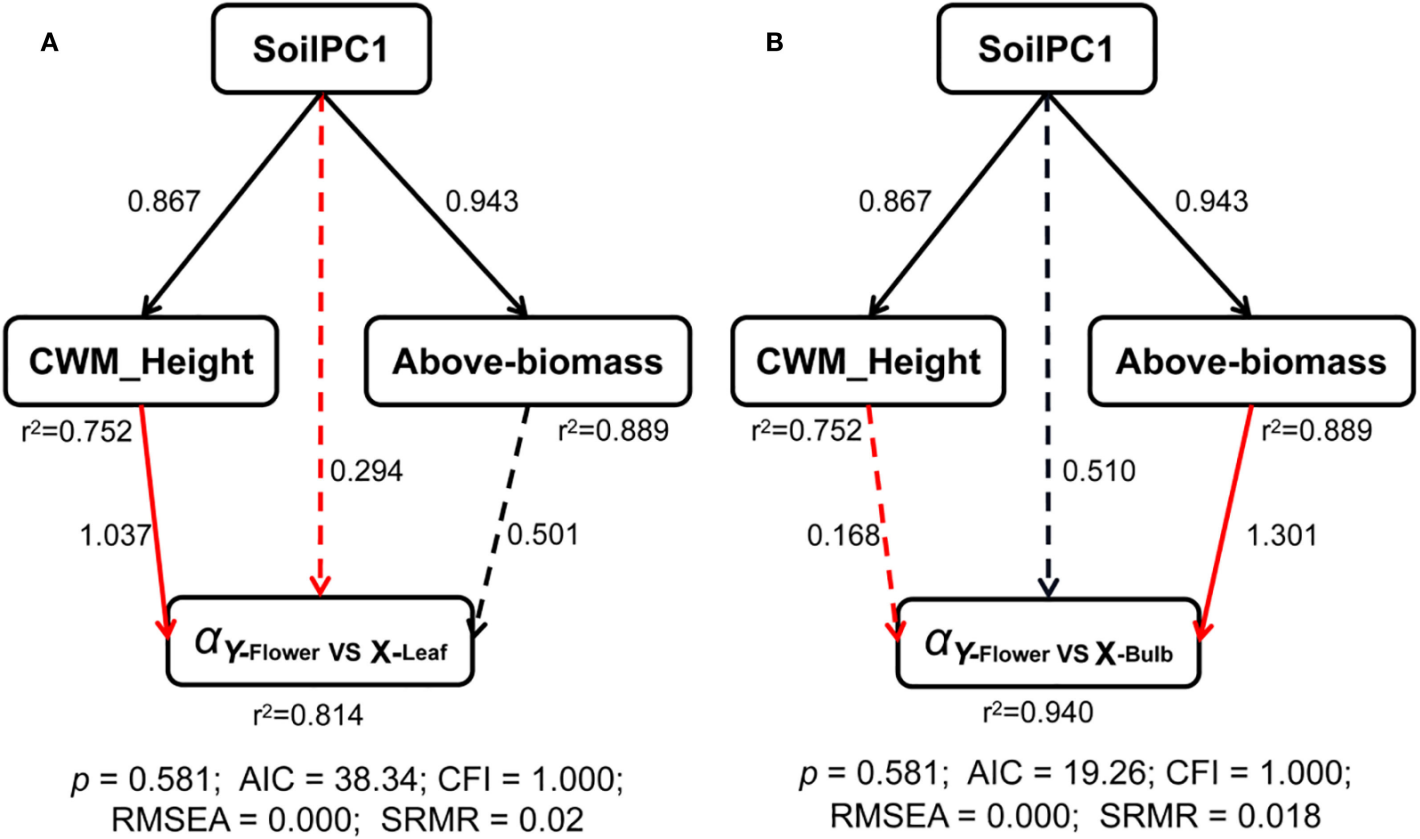
Structural equation models of driving mechanisms of multiple factors on scaling exponents of **(A)** leaf *vs* flower and **(B)** bulb *vs* flower. Black and red solid arrows represent significant (P < 0.05) positive and negative paths, respectively. Dashed black and red arrows represent non-significant (P > 0.05) positive and negative paths, respectively. Numbers represent the standardized path coefficients. The r^2^ represents the proportion of explained variance for exponents.

The results of the structural equation model showed that the direct effect of soilPC1 on the leaf-flower scaling exponents was not significant, but there was a significant positive effect on the plant community-weighted height, i.e. the plant community-weighted height increased as soil salinity decreased, but the community-weighted height had a significant negative effect on the leaf-flower scaling exponents, and thus there was an indirect negative effect of soilPC1 on the leaf-flower scaling exponents. SoilPC1 and plant community-weighted height together explained 81% of the variation in leaf-flower scaling exponents (**Figure 4A**). Similarly, soilPC1 indirectly influenced the scaling exponents between bulbs and flowers by influencing the aboveground biomass of the community, explaining 94% of the variance (**Figure 4B**).

## Discussion

### Changes in soil salinity gradients and plant communities

Soil salinization is a synergistic process of salt accumulation and nutrient loss. Studies have shown that salinity indicators (e.g. pH) are significantly negatively correlated with soil nutrients (organic carbon, nitrogen and phosphorus) in several ecosystems (Corwin, 2021; Garcia-Franco et al., 2021; Huang et al., 2024). Principal component analysis (PCA) further indicated that pH and EC could be the core indicators of soil degradation on the salinization gradient, while organic carbon and nitrogen and phosphorus became sensitive parameters for nutrient loss. The PCA showed that the salinity indicators (pH and EC) showed significant negative correlation with the nutrient indicators (organic carbon, nitrogen and phosphorus) (**Figure 1**), which revealed the close coupling between soil degradation and nutrient loss in the salinization process. This result is consistent with the general pattern of saline grassland degradation in the global scale (Pan et al., 2013; Heng et al., 2022; Li et al., 2022). For example, in saline soils in western Jilin Province, organic matter and quick potassium content decreased significantly with increasing sodium ion (Na^+^) content and EC, while total phosphorus content remained at a low level for a long time (Cao et al., 2021). This negative correlation can be explained by the following mechanism: ion competition and nutrient fixation: under high salinity conditions, Na^+^ displaces nutrients such as calcium (Ca^2+^) and magnesium (Mg^2+^) in the soil colloid by cation exchange, leading to their leaching or fixation (Zhou et al., 2023). Salt reduces organic matter mineralization and nutrient release by increasing soil osmotic pressure and toxicity and inhibiting microbial decomposition functions (Zhang et al., 2015; Chen et al., 2024). Experiments on the improvement of saline soils showed that the organic matter content of unimproved soils was only 50% of that of improved soils, verifying the negative effect of salts on soil carbon and nitrogen cycling (Xiao et al., 2025). Salinization leads to soil sloughing and reduced porosity, further hindering nutrient uptake by the root system (Garcia-Franco et al., 2021). The process forms a positive feedback of ‘salt accumulation-nutrient loss-soil degradation’, which ultimately leads to a systematic decline in soil fertility.

Natural environmental gradients shape patterns of plant diversity and are important factors influencing plant community dynamics. For example, along large-scale gradients (latitude, elevation), biodiversity tends to increase with energy, temperature and environmental stability, while within regional scales, such as degradation gradients and soil nutrient gradients, also significantly influence plant community structure and function (Zhou et al., 2021; Heng et al., 2022; Wu et al., 2024; Yang et al., 2025). We showed that plant community biomass and diversity decreased significantly with increasing salinization, while community variability increased (**Figure 2**). Salinization not only directly stresses plant physiology, but also affects community biomass and diversity by changing community structure and resource competition (Zhang et al., 2015). This result is consistent with studies in saline grassland, where salinization led to a large reduction in microbial community diversity (Heng et al., 2022). This change may be related to the fact that salinity inhibits plant photosynthesis and water uptake, leading to a decrease in overall community productivity (Corwin, 2021). At the same time, nutrient deprivation increases competition between species, and only a few salt-tolerant species can maintain population stability through phenotypic plasticity (e.g., reduced individual size, increased reproductive investment) (Tang et al., 2022). On the salinity gradient, local differences in soil physicochemical properties (e.g. pH and salinity patchy distribution) led to increased community variability (Zhang et al., 2015).

### Biomass allocation strategy for *Allium ramosum* in response to soil gradient

There was no significant trend observed in the biomass or stability of *Allium ramosum* populations along the soil gradient (**Figure 2e**). This phenomenon may be attributed to the physiological plasticity exhibited by *Allium ramosum*. Individuals within the species demonstrate a capacity to adapt to stressful environments through a reduction in body size, which leads to a decrease in resource requirements, and an increase in phenotypic variation, which can be considered a non-linear response (Huang et al., 2019; Ren et al., 2020; Matesanz et al., 2021). The impact of soil features on *Allium ramosum* populations may also be influenced by species interactions within the community. Our results demonstrated that individual size, plant height and the size of organs of *Allium ramosum* increased significantly with soil gradient (**Figure 2**, **Supplementary Figure S2**). These correlations indicate that natural selection may have prevented independent evolution of traits, suggesting a need for coordination among traits contributing to the same function (He et al., 2020; Zhou et al., 2022; Bin et al., 2024). The observed correlations among these traits can be regarded as a facet of the broader coordination between traits and organs in plants (Matesanz et al., 2021; Sanchez-Bermejo et al., 2023). Furthermore, in accordance with other studies, environmental stress did not modify the allometric distribution of biomass among organs, yet the allometric indices and constants exhibited a significant trend with soil salinity (Eziz et al., 2017; Peng et al., 2022). Growth and reproduction represent two of the most fundamental processes in plants, with the biomass of leaves, stems and roots determining the ability to capture light and access soil resources to provide photosynthetic products and nutrients for reproduction (Poorter et al., 2012; Yan et al., 2016; White et al., 2022). *Allium ramosum* exhibit an adaptive strategy for biomass allocation priority across salinity gradients. As soil salinity increase, the allocation of biomass to sexual reproductive organs (e.g. flowers) exceeds that allocated to nutritive organs (leaves, bulbs) (**Figure 3**).

Based on a field study conducted in the Sugan Lake wetland on the Qinghai-Tibet Plateau. Soil salinity conditions across three distinct habitats: inland salt marsh, oasis wetland, and seasonal river wetland significantly influenced *Saussurea salsa* biomass allocation and morphology. Under high salinity, plants developed large, thick leaves with low specific leaf area (SLA) and formed roots with moderate diameter and length, allocating minimal biomass to roots to mitigate water stress and ion toxicity by enhancing water storage in leaves and reducing root exposure (Li et al., 2021). This strategic allocation of biomass ensures the survival of offspring by increasing reproductive output under stressful conditions (Zhang et al., 2021a; Van de Walle et al., 2023; Stott et al., 2024). This variation in the strategy for biomass allocation is also subject to change in response to climate change and across large climatic and soil gradients, where increased resource stress (e.g. drought, low soil nutrient content) is experienced. *Artemisia* spp. exhibit increased biomass for reproduction allocation and decreased biomass allocation to leaves (Tsogtsaikhan et al., 2025). The decrease in leaves may be due to individual size limitation and reduced water consumption from transpiration, since more saline soils have poorer water-holding capacity (Zhou et al., 2023). However, it is important to note that the results of structural equation modelling suggest that this allocation pattern does not constitute a direct response to soil features, but is mediated through changes in community above-ground biomass and structure (**Figure 4**). The effect of soil salinization on the pattern of biological allocation between leaves and flowers of *Allium ramosum* was found to be indirectly influenced by affecting the community-weighted height of the plant. The decrease in community weighted height was attributed to salinization, resulting in the replacement of dominant species with low salt-tolerant species (Janousek and Folger, 2014; Token et al., 2022). At higher community heights, light competition is increased and plants allocate more biomass to growth, favoring plant extension for more light (Xiao et al., 2021). One reason why plants allocate fewer resources to reproduction may be mechanical limitations of mechanical support when plants are taller (West et al., 1999). According to Corner’s rules, larger inflorescences require thicker stems to support them (Fajardo et al., 2020). Hence, for *Allium ramosum*, taller stems is required so that the flowers can reach a sufficient height to favor pollination and fruit set. If plants devote more biomass to producing taller and thicker stems, this can lead to a less competitive plant, with stems being considered luxury organs (Xiao et al., 2021), especially in *Allium ramosum* plants, which only serve to support the inflorescence. Thus, as the community weighted height of the plant increases, the plant tends to allocate more biomass to leaves rather than flowers (**Figure 4a**).

It is also noteworthy that *Allium ramosum* is a perennial plant and that there will be a trade-off between storage and reproduction (Zhang et al., 2022). Our results also indicated that as soil salinity increased, plants similarly allocated more biomass to reproduction than to storage (**Figure 4b**). This strategy may reduce competition for light resources due to declining biomass in the plant community, prompting *Allium ramosum* to divert resources to reproduction in order to extend their dispersal advantage (Bonser, 2013). Conversely, in communities with less saline soils, increased aboveground biomass caused plants to allocate resources inside the storage organ - bulbs, to allow plants to have larger plants in the next growing season, as bulb size was significantly correlated with individual plant size (Howard and Cellinese, 2020; Ashagrie et al., 2021).

Notably, phosphorus has been found to be an essential element for plants, influencing the entire reproductive process, from bud differentiation to seed maturity (Fortier and Wright, 2021; Velez-Mora et al., 2024). At the intraspecific level, P-limited conditions resulted in later flowering onset, shorter individual flowering duration, and reduced flower/inflorescence production per plant. Interspecifically, species adapted to P-limited environments exhibited earlier flowering onset, longer seed stalks and panicles, but also shorter flowering periods and fewer flowers per plant (Wang et al., 2022). Critically, P limitation consistently constrained investment in sexual reproduction (e.g., reduced flower production, shorter flowering periods), potentially impairing dispersal capacity. Significant confounding effects of soil pH and moisture were also revealed which covaried with nutrient regimes—on reproductive traits, complicating the interpretation of N:P effects (Wang et al., 2022).

Based on the analysis of 599 Eurasian herbaceous sites, phosphorus (P) limitation (indicated by high N:P ratios in plant biomass) strongly influences plant reproductive strategies, with significant implications for endangered species. Plants in P-limited communities exhibit markedly reduced investment in sexual reproduction compared to nitrogen (N)-limited communities, manifested through lower seed production, diminished seed mass, shorter flowering periods, delayed flowering onset, and greater reliance on vegetative propagation and perennial lifespans (Fujita et al., 2014).

The experimental study investigated how absolute and relative nitrogen (N) and phosphorus (P) supply affect sexual reproduction traits in a common grass (*Holcus lanatus*) and an endangered forb (*Parnassia palustris*) demonstrated that the effect of N:P supply ratio on sexual reproduction investment is critically dependent on the absolute nutrient supply level: at low absolute nutrient supply, N:P ratio had minimal impact on reproduction traits, whereas at high absolute nutrient supply, a high N:P ratio (indicating low relative P availability) significantly reduced investment in sexual reproduction for *H. lanatus* (Wang et al., 2019). Low relative P-supply (high N:P ratio) restricted the positive response of sexual reproduction traits to increased absolute nutrient supply, essentially limiting the potential benefits of higher nutrient availability. While data for the endangered *P. palustris* were limited due to high mortality, its survival patterns mirrored the reproduction response of *H. lanatus*, suggesting similar constraints under low relative P-supply at high nutrient levels (Wang et al., 2019). This is consistent with our results that, as a perennial nondominant species, reproduction of *Allium ramosum* may be more strongly limited by phosphorus along the soil gradient (**Figure 1**, **Supplementary Table S1**).

The strategy for biomass allocation at the plant community level also produces adaptive variation in response to environmental change (Fant and Ghedini, 2024; Wu et al., 2024). A strategy for biomass allocation prioritizing survival is favored in arid environments, where more biomass is allocated to the root system for water, whereas investment in the above-ground fraction is prioritized in wetter areas with better soil nutrient (Wu et al., 2024). Global-scale studies indicate that the species composition and diversity of plant communities can buffer the effects of environmental gradients on biomass allocation (Skarpaas et al., 2016; Dolezal et al., 2021). For example, species turnover reduces the degree of variability in the overall allocation strategy of the community through functional trait complementarities (Zhou et al., 2021; Fang et al., 2023; Krak et al., 2025).

Recent studies of biomass allocation patterns have demonstrated considerable variation in this ratio across diverse environmental contexts (Chen et al., 2021; Beccari and Carmona, 2024; Dolezal et al., 2024). However, the majority of these studies have been conducted on individual plants, focusing exclusively on the abiotic environment and species traits, without considering the interactive dynamics among species within communities (Zhang et al., 2021a; Zhou et al., 2022; Krak et al., 2025; Tsogtsaikhan et al., 2025). Consequently, our understanding of how plants respond to the combined effects of soil salinity stress and species competition remains limited. The findings of this study contradict traditional, simplified models of direct soil-plant response and underscore the critical role of community-scale processes in individual adaptation strategies. At the community level, interspecific roles and environmental stresses may interact, complicating the individual to community level scaling transitions (Zhou et al., 2021; Fant and Ghedini, 2024). Consequently, we would like to propose that future studies include more characteristics of plant communities and populations when analyzing strategies for biomass allocation in response to environmental gradients or environmental changes.

## Conclusion

In this study, we investigated the biomass allocation patterns of various plant communities and *Allium ramosum* within these communities across a soil gradient. The results demonstrated that plant community diversity, community weighted height and aboveground biomass increased with soil gradient. Concurrently, the allometric exponent between leaf-flower and bulb-flower biomass exhibited a significant decreasing trend, with a greater allocation of biomass to sexual reproductive organs (flowers) than to vegetative organs (leaves and bulbs). The biomass allocation strategy of *Allium ramosum* was influenced by a decrease in community weighted height driven by soil salinization (reduced light competition) and changes in community aboveground biomass (competitive pressure for resources). These results highlight the influence of soil conditions and plant communities on plant life history strategies.

## Data Availability

The original contributions presented in the study are included in the article/**Supplementary Material**. Further inquiries can be directed to the corresponding author.
