# Supplementary material for: Soil and plant communities co-regulating plant biomass allocation patterns along a saline-alkali gradient, case study of Allium ramosum in Songnen Grassland, Northeast China
Source: Front Plant Sci. 2025 Sep 24;16:1627304. doi: 10.3389/fpls.2025.1627304 (PMC12504303; doi:10.3389/fpls.2025.1627304)

**Supporting information:**

| Soil character | Dim.1 | Dim.2 | Dim.3 | Dim.4 | Dim.5 |
| --- | --- | --- | --- | --- | --- |
| SOC | 0.898 | -0.163 | -0.092 | 0.392 | 0.053 |
| pH | -0.875 | 0.383 | 0.196 | 0.123 | -0.032 |
| EC | -0.840 | 0.438 | 0.239 | 0.111 | 0.086 |
| TN | 0.952 | 0.218 | 0.109 | -0.036 | 0.149 |
| TP | 0.759 | 0.591 | -0.244 | -0.035 | 0.084 |
| NP | 0.856 | -0.293 | 0.407 | -0.055 | 0.110 |
| AN | 0.914 | 0.295 | 0.048 | -0.095 | -0.027 |
| AP | 0.912 | 0.192 | 0.160 | 0.048 | -0.306 |
| Cumulative of  variation % | 76.975 | 89.107 | 93.715 | 96.189 | 98.032 |

Table S1. Correlation of each soil characters with the five principal axes of principal component analysis.

Table S2. Contribution of each soil indicator with the five principal axes of principal component analysis.

|  | Dim.1 | Dim.2 | Dim.3 | Dim.4 | Dim.5 |
| --- | --- | --- | --- | --- | --- |
| SOC | 13.094 | 2.735 | 2.302 | 77.651 | 1.939 |
| pH | 12.429 | 15.081 | 10.456 | 7.641 | 0.675 |
| EC | 11.464 | 19.746 | 15.539 | 6.181 | 5.002 |
| TN | 14.727 | 4.893 | 3.201 | 0.665 | 15.161 |
| TP | 9.346 | 35.970 | 16.091 | 0.636 | 4.759 |
| NP | 11.885 | 8.85 | 44.891 | 1.522 | 8.262 |
| AN | 13.553 | 8.945 | 0.612 | 4.55 | 0.481 |
| AP | 13.502 | 3.779 | 6.906 | 1.155 | 63.721 |

Table S3. Scaling exponents of biomass partitioning among organs of wild leeks in plant communities at different sites. ***p< 0.001; **p< 0.01; *p< 0.05.


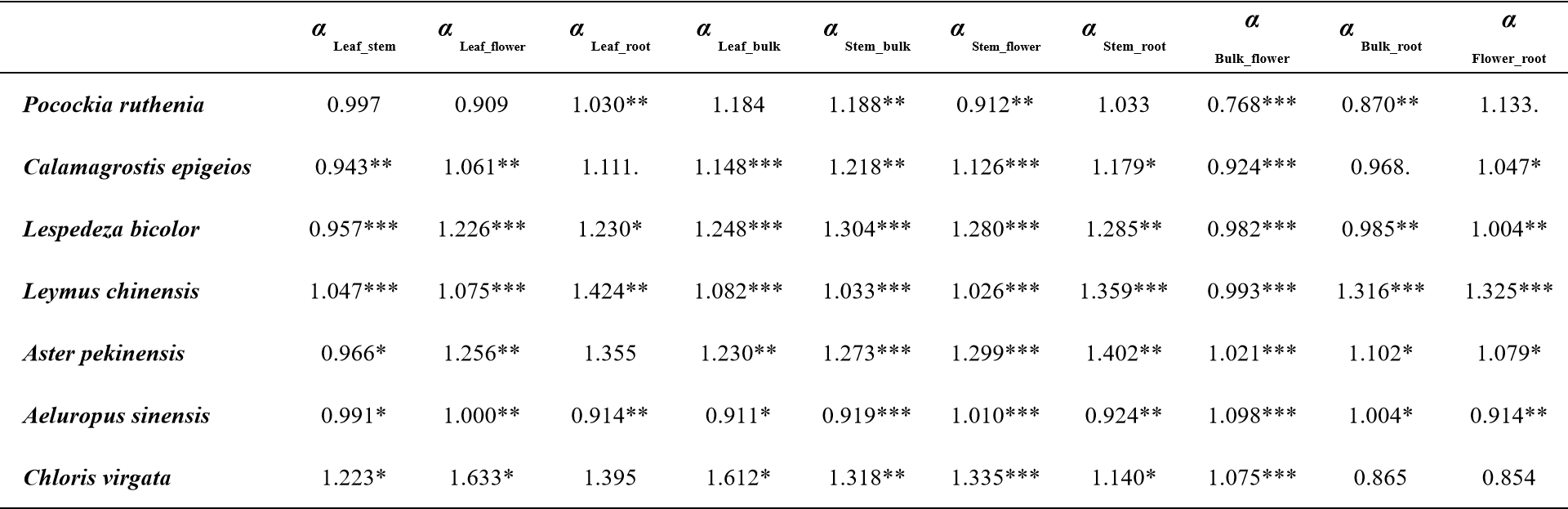


Figure S1. Relationship between community α diversity and above-ground biomass.


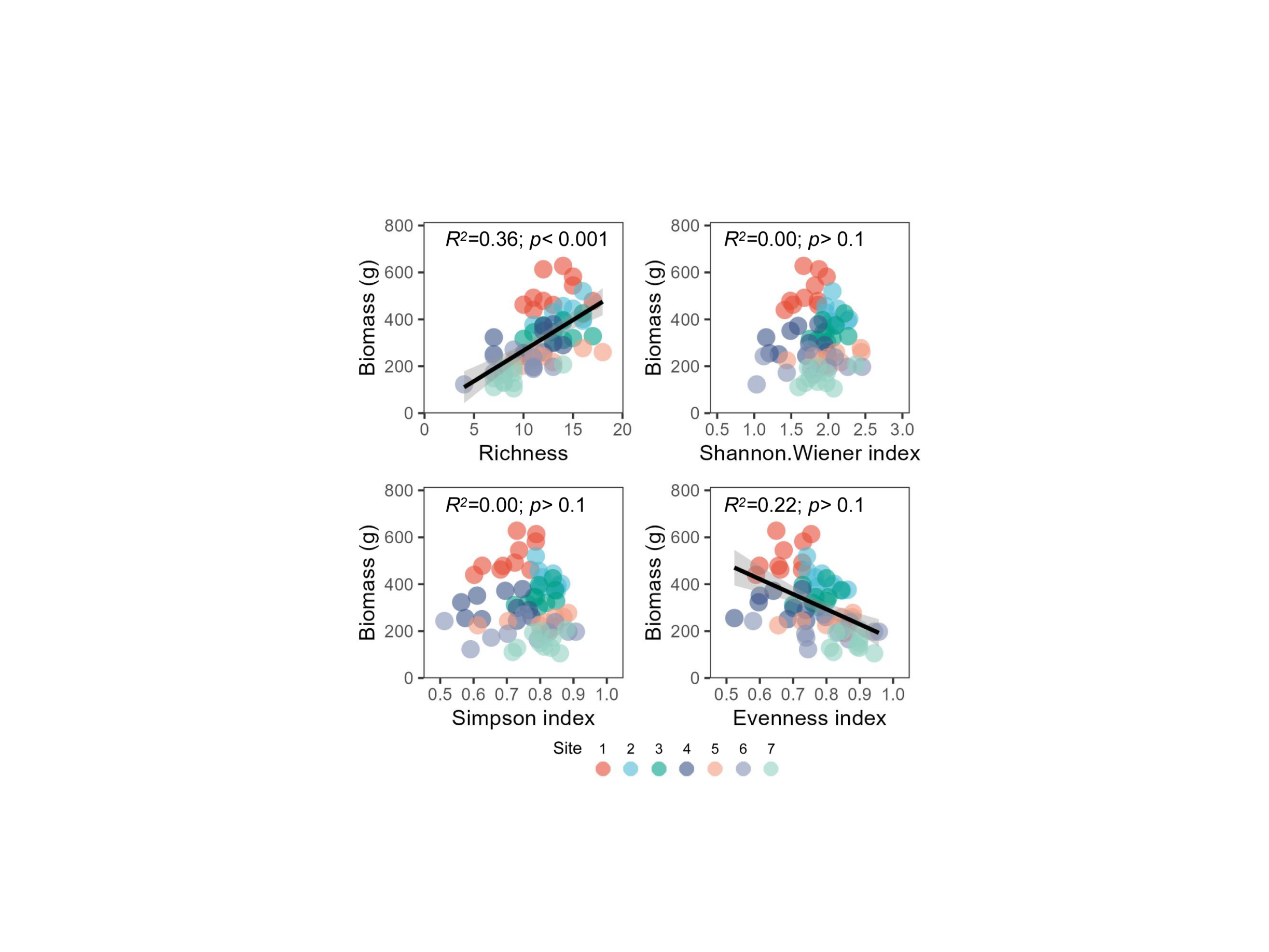


Figure S2. Relationships between plant height, organ size and soilPC1 , above-ground biomass.


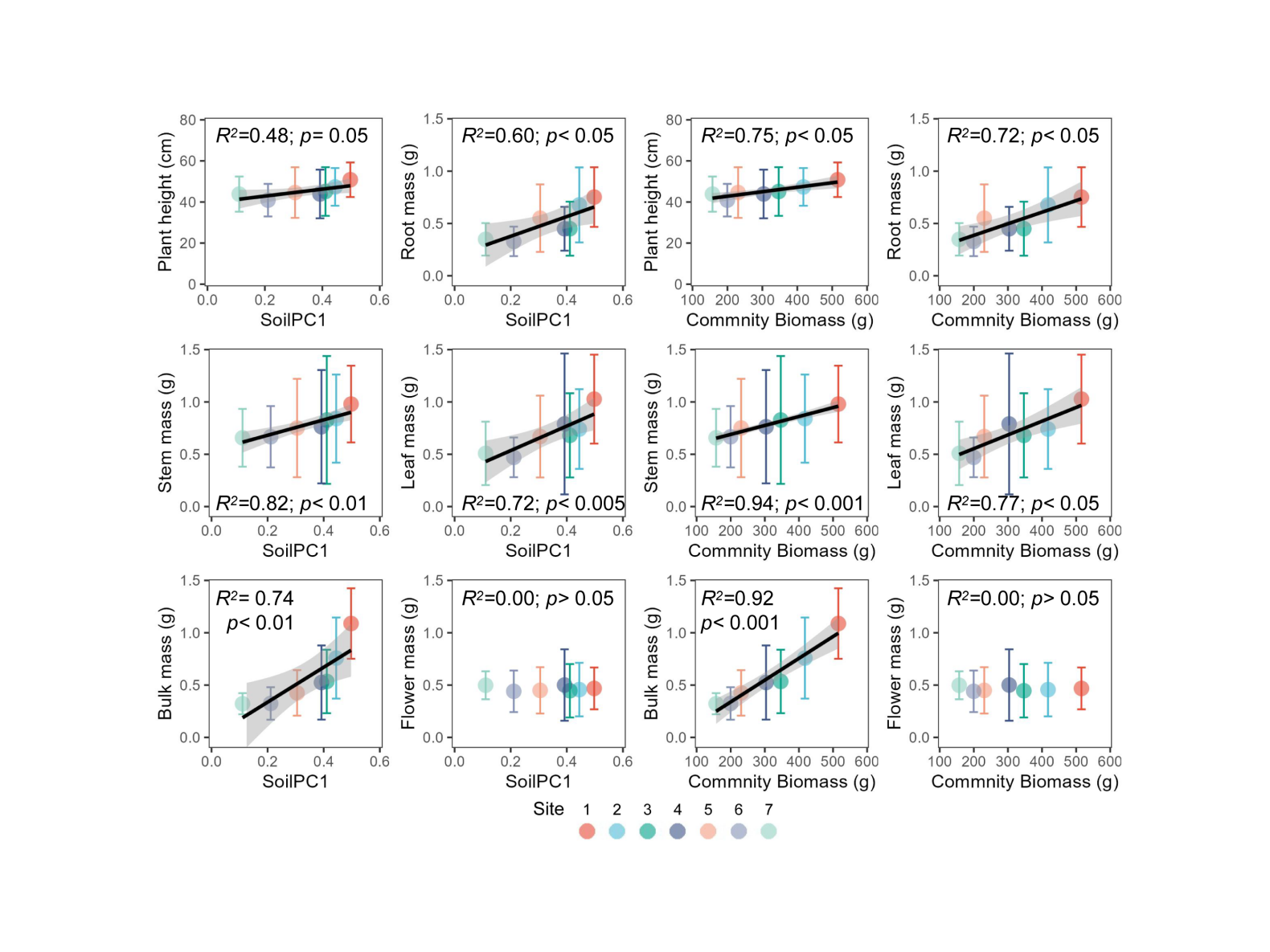


Figure S3. Relationships between scaling exponents of each organs and soilPC1 , above-ground biomass.


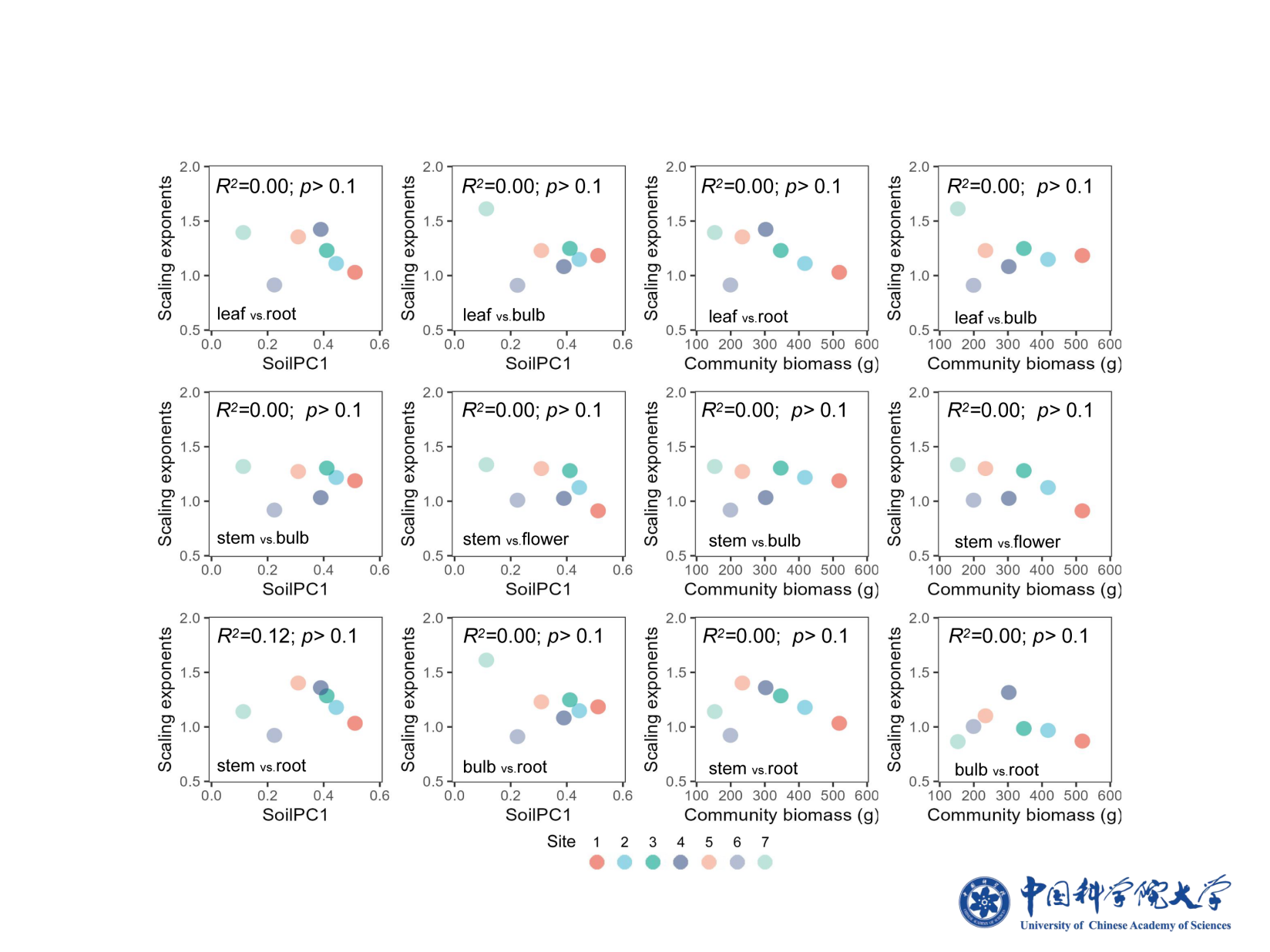


Figure S4. Relationship between SoilPC1 and population density.


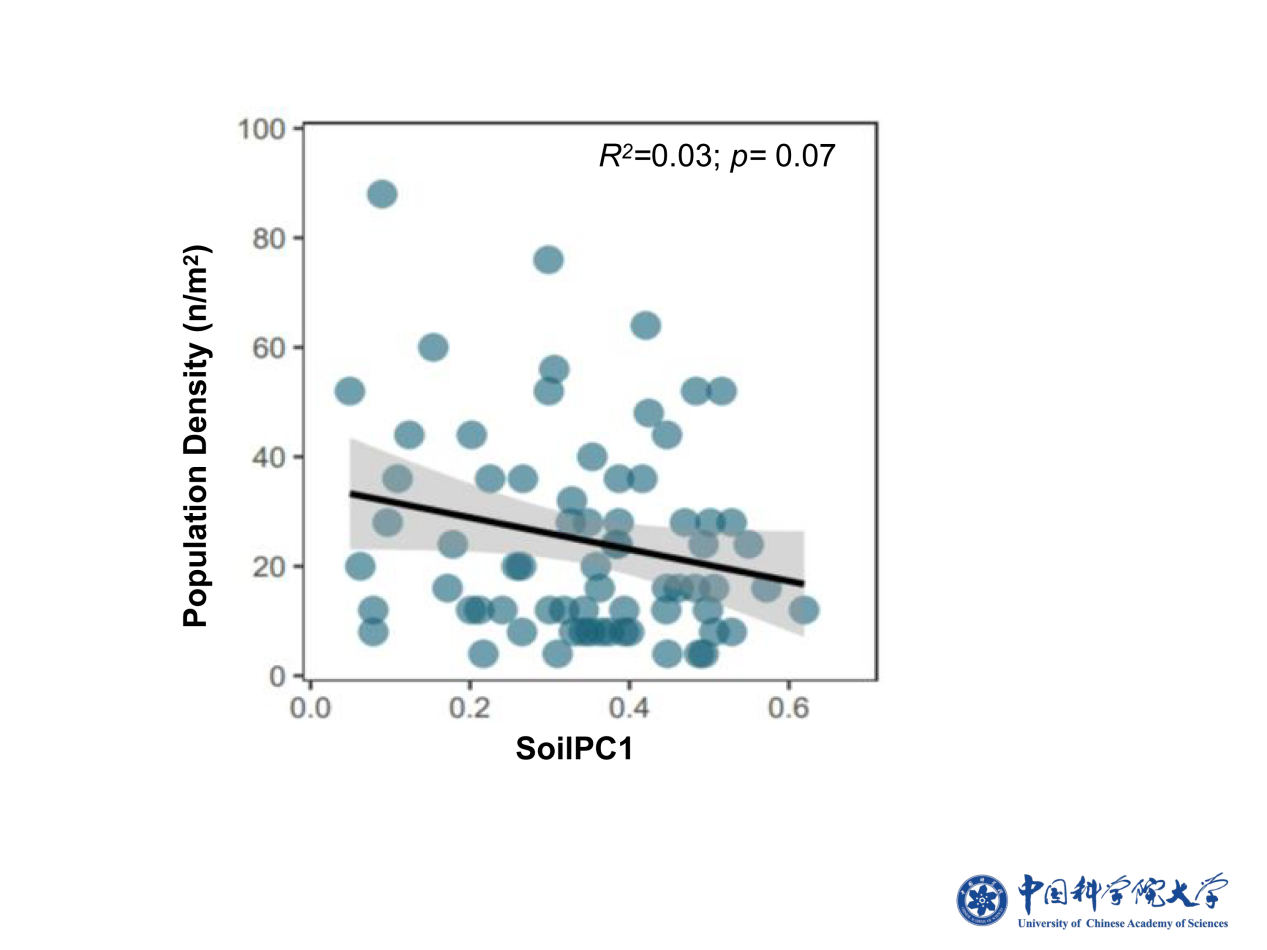

Supplement: Supplementary file 1 [file DataSheet1.docx]
